# Efficacy and safety of anti-interleukin-1 therapeutics in the treatment of knee osteoarthritis: a systematic review and meta-analysis of randomized controlled trials

**DOI:** 10.1186/s13018-023-03590-2

**Published:** 2023-02-13

**Authors:** Lizhi Yu, Raoshan Luo, Gang Qin, Qinyan Zhang, Weiming Liang

**Affiliations:** The First Affiliated Hospital of Guangxi University of Science and Technology, Guangxi University of Science and Technology, 124 Yuejin Road, Liuzhou, 545001 Guangxi Province China

**Keywords:** Anti-interleukin-1 therapeutics, Meta-analysis, IL-1 antibodies, Interleukin-1 receptor antagonist, IL-1 inhibitors

## Abstract

**Objective:**

We aimed to evaluate the efficacy and safety of anti-interleukin-1 therapeutics, including IL-1 antibodies, interleukin-1 receptor antagonists (IL-1 Ras) and IL-1 inhibitors, for knee osteoarthritis (KOA) treatment.

**Methods:**

Databases (Medline, Embase, Web of Science and CENTRAL) and ClinicalTrials.gov were systematically searched for randomized controlled trials (RCTs) of anti-interleukin-1 therapeutics from inception to August 31, 2022. The outcomes were the mean change in pain and function scores and the risk of adverse effects (AEs).

**Results:**

In the 12 studies included, anti-interleukin-1 therapeutics were superior to placebo in terms of pain relief (standardized mean difference [SMD] =  − 0.38, 95% confidence interval [CI] =  − 1.82 to − 0.40, *p* < 0.001, *I*^2^ = 77%) and functional improvement (SMD =  − 1.11, 95% CI =  − 1.82 to − 0.40, *p* = 0.002, *I*^2^ = 96%). The incidence of any AE (risk ratio [RR] = 1.02, 95% CI = 0.88–1.18, *p* < 0.001, *I*2 = 76%) was higher following treatment with anti-interleukin-1 therapeutics than placebo, while no significant difference was found in the incidence of serious AEs (SAEs) or discontinuations due to AEs. Subgroup analyses showed that IL-1 antibodies and the IL-1 inhibitor provided pain relief (IL-1 antibodies: SMD =  − 0.61, 95% CI =  − 0.92 to − 0.31, *p* < 0.001; IL-1 inhibitor: SMD =  − 0.39, 95% CI =  − 0.72 to − 0.06, *p* = 0.02, *I*2 = 74.0%) and functional improvement (IL-1 antibodies: SMD =  − 1.75, 95% CI =  − 2.10 to − 1.40, *p* < 0.001; IL-1 inhibitor: SMD =  − 0.28, 95% CI =  − 0.83 to 0.27, *p* = 0.31, *I*^2^ = 88%) superior to those of placebo, whereas IL-1 Ras did not. However, the IL-1 inhibitor increased the incidence of any AE (RR = 1.35, 95% CI = 0.92–1.98, *p* < 0.001, *I*^2^ = 85%) but not the risk of SAEs or discontinuations due to AEs. IL-1 antibodies and IL-1 Ras showed no difference in safety compared with placebo.

**Conclusions:**

Anti-interleukin-1 therapeutics could relieve OA-related pain and improve function, but is probably associated with an increased risk of adverse events. Specially, IL-1 antibodies and an IL-1 inhibitor could relieve OA-related pain and improve function, whereas IL-1 Ras could not. IL-1 antibodies and IL-1 Ras were relatively safe options, but IL-1 inhibitors were associated with safety concerns.

## Introduction

Osteoarthritis (OA) is a whole-joint disease in which all of the components of the joint are affected [1]. OA is the most common joint disease, with more than 240 million people suffering varying degrees of OA worldwide, and the knee joint (knee OA, KOA) is the most commonly affected joint [2, 3]. The main clinical symptoms of KOA are pain, stiffness, and limited mobility, which are associated with the inflammation of the knee joint and greatly affects the patient’s quality of life [4–6]. Patients with end-stage KOA can be well treated with knee replacement [7], but the same treatment is unacceptable for early-stage KOA or young and middle-aged KOA patients. Thus, conservative nonsurgical interventions are proposed to treat painful joints [8–10]. At present, nonsurgical interventions are mainly used to relieve clinical symptoms, improve joint function, and slow down the degeneration of intra-articular structures to avoid or delay joint replacement surgery [11–15]. Nonsurgical treatment options for KOA include a wide variety of drugs, including nonsteroidal anti-inflammatory drugs (NSAIDs), opioids, steroids, and hyaluronic acid (HA), as well as exercise therapy and weight loss, but the results are not satisfactory [13, 16–23]. Moreover, NSAIDs and opioids are poorly tolerated in many patients, and the safety profile of long-term therapy with NSAIDs or opioids is concerning [17, 18, 24, 25]. Therefore, a new KOA treatment direction is urgently needed.

With the further study of the pathological mechanism of OA, an increasing number of new targets have been discovered and have become the focus of recent pharmaprojects. Inflammatory cytokines, such as interleukin (IL), tumour necrosis factor (TNF), and nerve growth factor (NGF), which are the key mediators that promote the pathophysiology of KOA, cannot be ignored in the occurrence of OA. It has been shown that inflammatory cytokines act as a signals that mimic chondrodegradation enzymes from chondrocytes [26]. IL-1 $\upbeta$ and tumour necrosis factor-α (TNF-α) are the key cytokines in the cartilage catabolic process [27–31]. Among the many ILs, IL-1α, IL-1β, and other IL-1 family members are the most highly profiled and have all been shown to be present in the synovial fluid and subchondral bone of OA patients [32–34]. IL-1β is involved in the pathogenesis of cartilage loss and destructive OA [34–36]. With the deepening of basic research, it has become an established fact that IL-1 triggers KOA; therefore, whether anti-IL-1 therapy could treat KOA has aroused great interest from researchers [37, 38].

The current anti-IL-1 therapeutics found in the available literature for KOA mainly consist of the following three types: IL-1 antibodies, IL-1 Ras, and IL-1 inhibitors [39–45]. The literature included in the current published meta-analysis is not comprehensive, as some drugs are missing or the latest research results are missing as they were not available at the time of publication, so the inconsistent efficacy and safety of anti-IL-1 therapeutics in KOA reported in the literature cannot comprehensively explain the advantages and disadvantages of anti-IL-1 therapeutics [46–48]. Therefore, the correct clinical treatment strategy may not be made by solely relying on the results of existing studies and there is a need to update the data on the efficacy and safety of anti-IL-1 therapeutics.

The purpose of this meta-analysis was to evaluate the efficacy and safety of anti-IL-1 therapeutics for KOA treatment. Pain and function scores as well as adverse events were evaluated in a meta-analysis of RCTs. We hypothesized that anti-IL-1 therapeutics would be more efficacious in terms of pain relief and functional improvement in the treatment of patients with KOA than control treatment, and anti-IL-1 therapeutics were relatively safe options.

## Methods

The present study was completed according to the Cochrane guidelines for issues related to the methodology of systematic reviews [49].

### Search strategy

We conducted a systematic literature search in Medline (1946 to August 31, 2022), Embase (1974 to August 31, 2022), Web of Science (1966 to August 31, 2022), and CENTRAL(1995 to August 31, 2022) to identify relevant studies. The search strategy was as follows: ((((((("lutikizumab" [Supplementary Concept]) OR (((ABT-981) OR (an anti-interleukin-1alpha and anti-interleukin-1beta dual variable domain immunoglobulin)) OR ("lutikizumab" [Supplementary Concept]))) OR ("Interleukin 1 Receptor Antagonist Protein"[Mesh])) OR ((((((((((((((IL1 Febrile Inhibitor) OR (Febrile Inhibitor, IL1)) OR (IL-1Ra)) OR (Urine-Derived IL1 Inhibitor)) OR (IL1 Inhibitor, Urine-Derived)) OR (Urine Derived IL1 Inhibitor)) OR (IL-1 Inhibitor, Urine)) OR (IL 1 Inhibitor, Urine)) OR (Urine IL-1 Inhibitor)) OR (Interleukin 1 Inhibitor, Urine)) OR (Antril)) OR (Kineret)) OR (Anakinra)))) OR (diacerein)) OR ("canakinumab" [Supplementary Concept])) AND (((((((((((Osteoarthritides) OR (Osteoarthrosis)) OR (Osteoarthroses)) OR (Arthritis, Degenerative)) OR (Arthritides, Degenerative)) OR (Degenerative Arthritides)) OR (Degenerative Arthritis)) OR (Arthrosis)) OR (Arthroses)) OR (Osteoarthrosis Deformans)) OR ("Osteoarthritis"[Mesh]))) AND (randomized controlled trial[Publication Type] OR randomized[Title/Abstract] OR placebo[Title/Abstract]).

We also manually checked the bibliographies of the identified articles, including relevant reviews and meta-analyses, to identify additional eligible studies. Furthermore, we searched three clinical trial registries (ClinicalTrials.gov, Controlled-trials.com, and Umin.ac.jp/ctr/index. The htm), as we allowed the inclusion of unpublished clinical studies.

### Selection criteria

We included studies in this systematic review and meta-analysis based on the following criteria: (1) patients: patients diagnosed with KOA based on the criteria described by the American College of Rheumatology; (2) intervention: treatment with anti-IL-1 therapeutics; (3) comparison: treatment with placebo, saline, or no treatment; (4) outcomes: at least 1 of the following outcomes: the Western Ontario and McMaster Universities Osteoarthritis Index (WOMAC) total score, WOMAC subscores (pain, function, and stiffness), the visual analogue scale (VAS) score for pain, the pain and function subitem scores of the Knee Injury and Osteoarthritis Outcome Score (KOOS), and adverse events (defined as local and systemic reactions such as pain, stiffness, swelling, dizziness, headache, nausea, or infection); and (5) studies: RCTs. The following studies will be excluded: (1) other documents included due to expansion of search scope, such as retrospective research, review, or meta-analysis; (2) non-knee joint; (3) failed to obtain outcome indicators; (4) small sample size: less than 5 participants in intervention arms; and (5) non-RCT.

### Selection of studies

EndNote (Version 20; Clarivate Analytics) was used to manage the selection of studies, including duplicate removal. Two reviewers (R.L. and Q.Z.) independently carried out the initial search, removed duplicate records, screened the titles and abstracts for relevance, and classified each study as included, excluded. We resolved disagreements by consensus. If no agreement was met, a third review author (G.Q.) acted as arbiter.

### Data extraction

Data were extracted by 2 reviewers (R.L. and Q.Z.), input into a standardized electronic form, and checked by a third reviewer (G.Q.). Disagreements were resolved through discussion before the analyses were performed. The following data were extracted: first author, year of publication, country, company, number of participants, age, sex, body mass index (BMI), severity of OA, intervention, method of administration, and outcome data. Predefined primary outcomes were WOMAC pain and function scores, the VAS score for pain, the pain and function subitem scores of the KOOS, any AE, serious AEs (SAEs), and discontinuations due to AEs. An AE that was life-threatening, disabling, led to hospitalization or death, or led to a birth defect or congenital anomaly was classified as a SAE. It was classified as discontinuation due to AEs when patient dropped out of the trial or patient was withdrawn from the trial at the judgement of the investigator due to any AE. When the same patients were reported in several publications, we retained only the latest study to avoid the duplication of information. Because of the different follow-up times of these identified studies, we pooled and calculated data from around a similar time frame. Since the shortest follow-up among these identified studies is 3 months, data from follow-up in the second or third month were merged.

### Risk of bias assessment

Two reviewers (R.L. and Q.Z.) used the Cochrane Risk of Bias tool to assess the risk of bias in the RCTs. Each study was reviewed and scored as having a high, low, or unclear risk of bias according to the following domains: random sequence generation, allocation concealment, blinding of participants and personnel, blinding of outcome assessment, incomplete outcome data, selective reporting, and other bias. Discrepancies between the reviewers were resolved by discussion until consensus was achieved.

### Data analysis and statistical methods

We analysed the results of the studies using RevMan 5.4 (Cochrane Collaboration, Oxford, UK). Results of dichotomous data were presented as risk ratios (RR) with the corresponding 95% confidence intervals (95% CI). An RR greater than 1.0 indicated a beneficial effect of anti-IL-1 therapeutics. Results of continuous data were presented as mean differences (MD) between the intervention and comparator groups with the corresponding 95% CIs. Since pain and function were measured by different scales, we calculated standardized mean differences (SMD) with the corresponding 95% CIs instead. For the calculation of SMD, we divided the MD by the standard deviation, resulting in a unit-less measure of treatment effect. SMDs less than zero indicated a beneficial effect of the anti-interleukin-1 therapeutics. As described by Cohen, an SMD of 0.2 indicates a small beneficial effect, 0.5 a medium effect, and 0.8 a large effect in favour of anti-interleukin-1 therapeutics [50]. Statistical heterogeneity was assessed using a standard chi-square test and was considered significant at *p* < 0.05. Pooled data were analysed using a random effects model because we assumed that there is heterogeneity caused by factors other than chance. The overall effect size is shown in forest plots. We stratified the analyses according to the mechanism of action to understand the effects of different anti-IL-1 therapeutics on pain and function and the AEs associated with treatment.

## Results

### Literature search

**Fig. 1:**
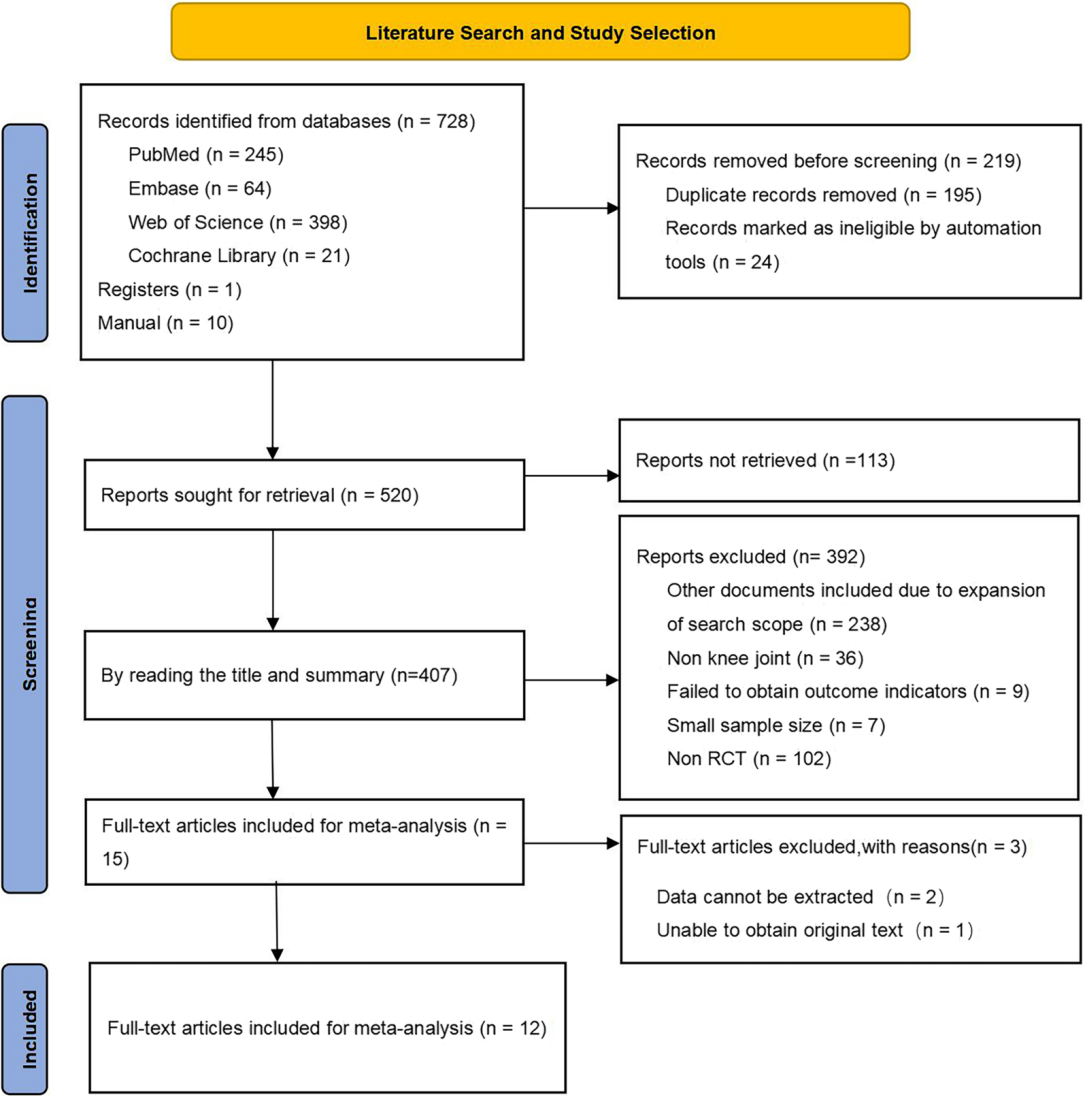
Flow chart of the literature search and study selection

Figure 1 shows the process of the study selection and inclusion. A total of 728 potential studies were identified with the initial search strategy. A total of 10 studies were obtained after the manual reference review, and one unpublished study was retrieved from ClinicalTrials.gov. After the examination of the titles and abstracts, 15 eligible studies were assessed for potential inclusion. After reviewing the full texts, 12 RCTs were included in the meta-analysis [51–62].

### Study characteristics

The detailed information of the included studies and the baseline characteristics of the included patients are presented in Tables 1 and 2, respectively. The anti-IL-1 therapeutics evaluated in the literature could be divided into the following three categories based on their mechanism of action. Placebo was used as control groups in all 12 studies, but only three of them clearly informed that physiological saline was used as placebo [53, 54, 61], and others had not explained what was used as placebo. The sample size of the studies ranged from 36 to 480, for a total of 2192 knees, including 1361 knees in the anti-IL-1 therapeutics group and 831 knees in the placebo group.

**Table 1:** Detailed information of the included studies

Study and year	Type	Country	Company	Medicine	Intervention	Follow-up, weeks	Group	Subgroup§	Outcomes‡
Fleischmann 2019 [51]	RCT	USA	AbbVie Inc	ABT981(IL-1 antibody)	SC, once every 2 weeks, lasting for 50 weeks	52	25 mg 100 mg 200 mg	Subgroup 1 Subgroup 2 Subgroup 3	①②⑥
Cohen 2011 [52]	RCT	USA	Amgen Inc	AMG 108 (IL-1 Ras)	SC, once every 4 weeks, lasting for 12 weeks IV, once every 4 weeks, lasting for 12 weeks SC, once every 4 weeks, lasting for 12 weeks	12	75 mg 300 mg 100 mg 300 mg 300 mg	Subgroup 1 Subgroup 2 Subgroup 3 Subgroup 4 Subgroup5	⑥
Baltzer 2009 [53]	Multicentre enter RCT	Germany	NA	Orthokine† (IL-1 Ras)	IA, twice a week, lasting for 3 weeks	26	NA	Subgroup 1	①②⑤⑥
Yang 2008 [54]	RCT	Netherlands	NA	Orthokine† (IL-1 Ras)	IA on days 0, 3, 7, 10, 14 and 21	48	NA	Subgroup 1	③④⑤
Wang 2017 [55]	RCT	USA	AbbVie Inc	ABT-981 (IL-1 antibody)	SC, once every 1 week, lasting for 8 weeks SC, once every 4 weeks, lasting for 12 weeks	16.7 18.1	0.3 mg/kg 1 mg/kg 3 mg/kg 3 mg/kg	Subgroup 1 Subgroup 2 Subgroup 3 Subgroup 4	⑥
Chevalier 2009 [56]	Multicentre enter RCT	France	Amgen Inc	Anakinra (IL-1 Ras)	Single IA	12	50 mg 150 mg	Subgroup 1 Subgroup 2	⑤⑥
Kosloski 2016 [57]	RCT	USA	AbbVie Inc	ABT-981(IL-1 antibody)	SC, once every 2 weeks, lasting for 8 weeks SC, once every 4 weeks, lasting for 12 weeks	18	0.3 mg/kg 1 mg/kg 3 mg/kg 3 mg/kg	Subgroup 1 Subgroup 2 Subgroup 3 Subgroup 4	⑥
NCT01160822 2012[58]	RCT	USA	Novartis Inc	Canakinumab (IL-1 antibody)	Single IA Single IA of canakinumab + oral placebo twice a day (control group: placebo + placebo), lasting for 12 weeks	18	150 mg 300 mg 600 mg 600 mg Canakinumab + placebo	Subgroup 1 Subgroup 2 Subgroup 3 Subgroup 4	⑥
Brahmachari 2009 [59]	RCT	India	NA	Diacerein(IL-1 inhibitor)	Oral, once a day for the first 10 days, 50 mg after meals, lasting for 8 weeks	12	NA	Subgroup 1	②⑤⑥
Pelletier 2000 [60]	Multicentre enter RCT	France	Les Laboratoires Negma	Diacerein(IL-1 inhibitor)	Oral twice a day for 16 weeks	12	50 mg 100 mg 150 mg	Subgroup 1 Subgroup 2 Subgroup 3	①②⑤⑥
Pham 2004 [61]	Multicentre enter RCT	France	NA	Diacerein(IL-1 inhibitor)	Oral twice a day for 12 weeks	48	NA	Subgroup 1	⑤⑥
Pavelka 2007 [62]	Multicentre enter RCT	Czech	TRB Chemedica and Glynn Brothers Chemicals	Diacerein(IL-1 inhibitor)	Oral twice a day for 12 weeks	24	NA	Subgroup 1	②⑥

^*^RCT randomized controlled trial; *SC* subcutaneous injection; *IV* intravenous injection; *IA* intra-articular injection; *WOMAC* Western Ontario and McMaster Universities Osteoarthritis Index; *KOOS* Knee Injury and Osteoarthritis Outcome Score; *VAS* visual analogue scale; and *NA* not available

Universities Osteoarthritis Index

^†^Orthokine: Orthokine is the trade name of autologous-conditioned serum (ACS)

^‡^Data extraction results of outcomes: ① WOMAC pain score; ② WOMAC function score; ③ KOOS pain score; ④ KOOS function score; ⑤ VAS score; and ⑥ adverse events

^§^Due to the limited number of studies, we named and grouped the research projects in different ways according to the original research

Baseline characteristics of studies included in the meta-analysis

**Table 2:** Baseline characteristics of the included patients*

										Kellgren and Lawrence grading scale, n (%)					
		Sample size, n		Female, n (%)		Age, y (M ± SD)		BMI, kg/m^2^ (M ± SD)		Treat		Control		Course of OA, y (M ± SD)	
Study and year	Subgroup	Treat	Control	Treat	Control	Treat	Control	Treat	Control	Grade II	Grade III	Grade II	Grade III	Treat	Control
Fleischmann 2019 [51]	Subgroup 1	89	85	63 (70.8)	52 (61.2)	61.6 ± 7.5	59.5 ± 8.9	28.7 ± 3.8	28.6 ± 3.6	57 (64.0)	32 (36.0)	53 (62.4)	32 (37.6)	7.6 ± 9.0	7.9 ± 8.0
	Subgroup 2	85		53 (62.4)		60.2 ± 8.2		29.0 ± 3.5		52 (61.2)	33 (38.8)			7.9 ± 8.7	
	Subgroup 3	88		57 (64.8)		59.1 ± 10.3		28.7 ± 3.5		56 (63.6)	32 (36.4)			8.7 ± 8.6	
Cohen 2011 [52]	Subgroup 1	12	16	9 (75)	10 (63)	62.3	60.8	30.9	30.4	4 (33)	5 (42)	4 (25)	10 (63)	10	9.6
	Subgroup 2	12		5 (42)		59.6		29.8		5 (42)	7 (58)			6.6	
	Subgroup 3	12		11 (92)		61.1		30.8		3 (25)	6 (50)			6.9	
	Subgroup 4	12		7 (58)		62.8		31.9		7 (58)	4 (33)			10.2	
	Subgroup5	80	80	54 (68)	54 (68)	61.3	60.1	32	31.9	40 (50)	39 (49)	30 (38)	46 (58)	6.1	6.1
Baltzer 2009 [53]	Subgroup 1	134	107	65 (48.5)	68 (63.6)	53.8 ± 12.2	60.3 ± 10.7	NA	NA	NA	NA	NA	NA	NA	NA
Yang 2008 [54]	Subgroup 1	73	67	49 (61)	43 (59)	54 ± 11	53 ± 11	27 ± 5	28 ± 14	NA	NA	NA	NA	NA	NA
Wang 2017 [55]	Subgroup 1	7	6	5 (71.4)	5 (83.3)	61.3 ± 5.1	60.0 ± 5.9	27.6 ± 4.4	28.4 ± 2.3	NA	NA	NA	NA	NA	NA
	Subgroup 2	7		5 (71.4)		62.6 ± 3.6		26.4 ± 1.1		NA	NA	NA	NA	NA	NA
	Subgroup 3	7		5 (71.4)		61.4 ± 5.0		27.3 ± 2.9		NA	NA	NA	NA	NA	NA
	Subgroup 4	7	2	7 (100)	2 (100)	60.0 ± 6.1	55.0 ± 1.4	29.3 ± 3.0	8.7 ± 0.5	NA	NA	NA	NA	NA	NA
Chevalier 2009 [56]	Subgroup 1	34	69	17 (50)	44 (64)	63.3 ± 9.8	62.2 ± 10.0	NA	NA	3 (4)	18 (53)	27 (39)	42 (61)	8.1 ± 9.8	6.0 ± 6.2
	Subgroup 2	67		46 (69)		62.6 ± 9.4		NA	NA	24 (37)	39 (58)			5.2 ± 5.7	
Kosloski 2016 [57]	Subgroup 1	7	8	24 (86)	7 (88)	61.3 ± 11.85	58.8 ± 9.63	NA	NA	NA	NA	NA	NA	NA	NA
	Subgroup 2	7						NA	NA	NA	NA	NA	NA	NA	NA
	Subgroup 3	7						NA	NA	NA	NA	NA	NA	NA	NA
	Subgroup 4	7						NA	NA	NA	NA	NA	NA	NA	NA
NCT01160822 2012	Subgroup 1	6	5	7 (88)	2 (40)	58.3 ± 12.79	57.8 ± 7.76	NA	NA	NA	NA	NA	NA	NA	NA
	Subgroup 2	7		4 (57.1)		61.0 ± 9.63		NA	NA	NA	NA	NA	NA	NA	NA
	Subgroup 3	6		2 (33.3)		64.2 ± 10.68		NA	NA	NA	NA	NA	NA	NA	NA
	Subgroup 4	45	47	31 (68.9)	31 (66)	61.4 ± 8.96	60.3 ± 9.71	NA	NA	NA	NA	NA	NA	NA	NA
Brahmachari 2009 [59]	Subgroup 1	28	27	26 (92.8)	20 (74)	45.5 ± 10.52	53 ± 11.85	25.3 ± 3.63	20 ± 3.70	10	18	12	15	3.5 ± 6.0	2.0 ± 3.3
Pelletier 2000 [60]	Subgroup 1	126	124	105 (83.3)	98 (79)	62.95 ± 8.41	64.5 ± 8.65	31.63 ± 5.50	31.05 ± 5.35	NA	NA	NA	NA	7.8 ± 7.18	8.0 ± 7.41
	Subgroup 2	110		83 (75.5)		64.22 ± 8.02		31.73 ± 6.21		NA	NA	NA	NA	8.1 ± 6.42	
	Subgroup 3	120		96 (80)		62.27 ± 10.18		30.99 ± 5.88		NA	NA	NA	NA	7.8 ± 6.99	
Pham 2004 [61]	Subgroup 1	85	85	59 (69.4)	52 (61.2)	64.5 ± 7.8	64.9 ± 7.7	NA	NA	12 (14)	66 (79)	19 (23)	60 (72)	NA	NA
Pavelka 2007 [62]	Subgroup 1	82	83	67 (81.7)	64 (77.1)	63.5 ± 8.39	63.8 ± 8.09	28.7 ± 4.1	29.1 ± 3.9	54 (65.9)	27 (32.9)	48 (57.8)	35 (42.2)	6.87 ± 6.16	6.13 ± 5.61

^*^BMI body mass index; *NA* not available; *M* mean; *SD* standard deviation; and continuous data are depicted in M when SD is not available

### Risk of bias

**Fig. 2:**
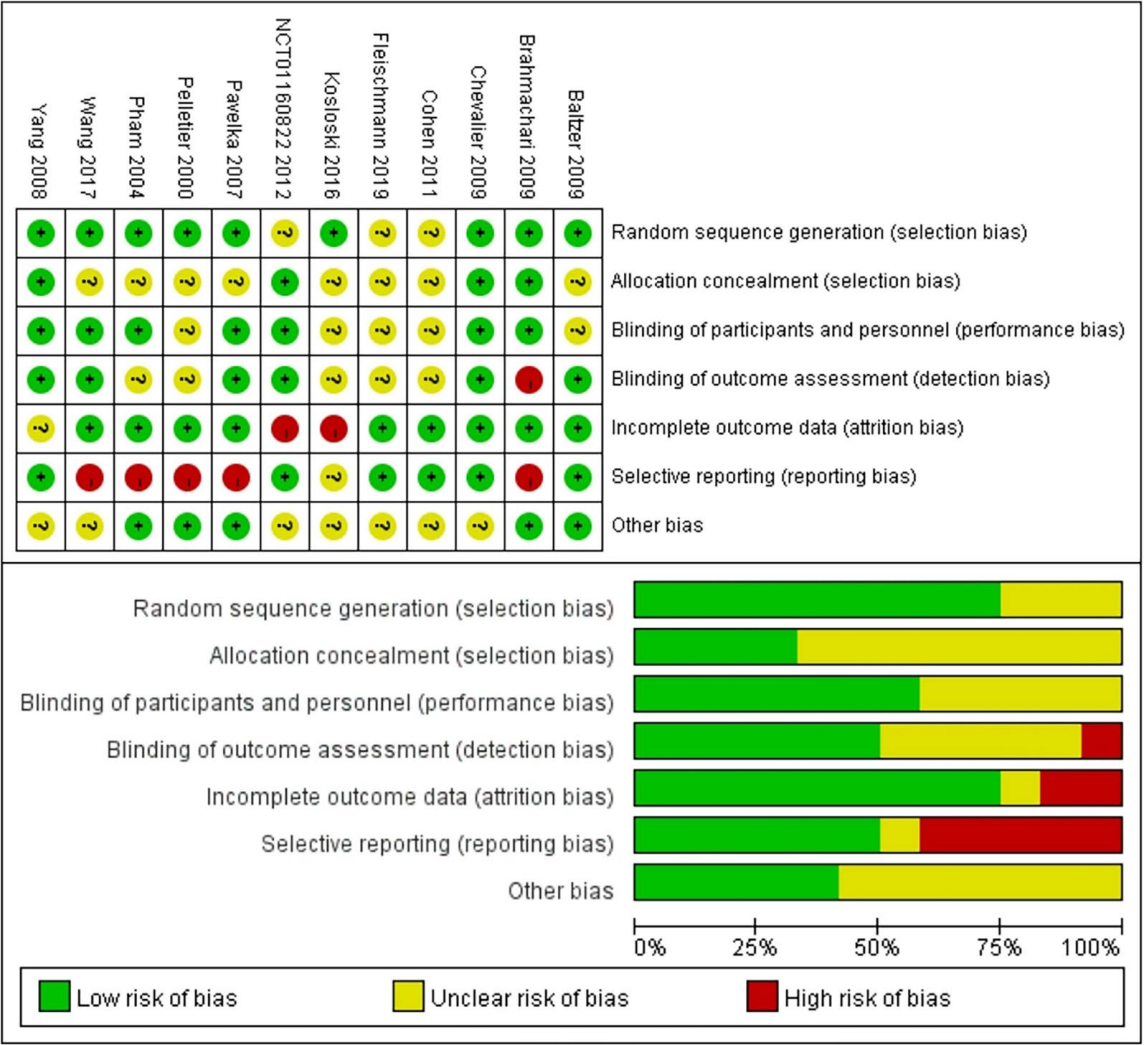
Risk of bias assessment for the included studies

The results of the risk of bias assessment are summarized in Fig. 2. Among the 12 studies, 7 studies were judged to have a high risk of bias [55, 57–62], and 5 were found to have a moderate risk of bias [51–54, 56]. An adequate randomized sequence was generated in 9 studies [53–57, 59–62], appropriate allocation concealment was reported in 4 studies [54, 56, 58, 59], the blinding of participants was clear in 7 studies [54–56, 58, 59, 61, 62], the blinding of outcome assessors was reported in 6 studies [53–56, 58, 62], outcome data were complete in 9 studies [51–53, 55, 56, 59–62], 6 studies had no selective reporting [51–54, 56, 58], and 5 studies had no other bias [53, 59–62].

### Knee pain scores

**Fig. 3:**
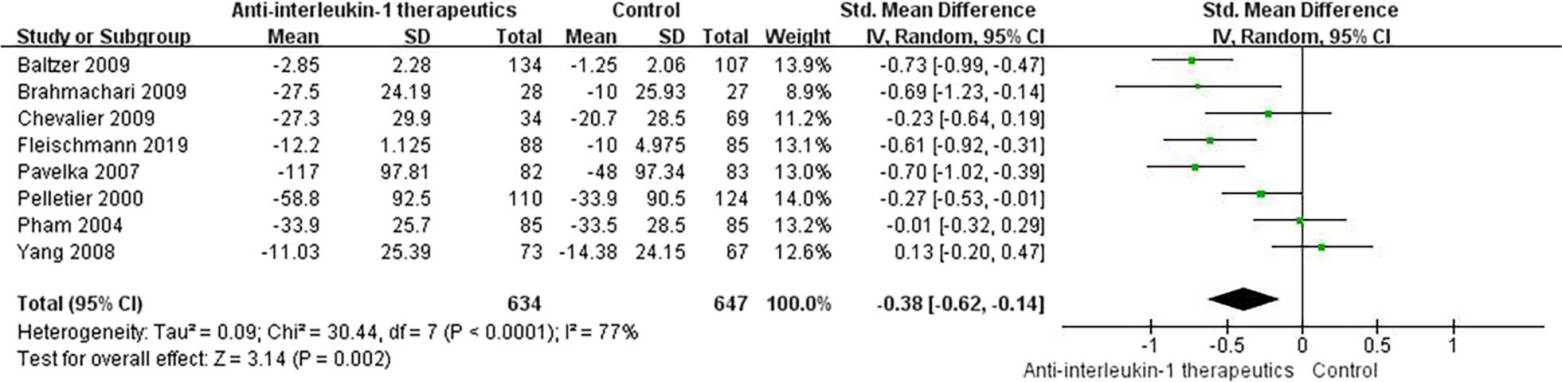
Knee pain score results

**Fig. 4:**
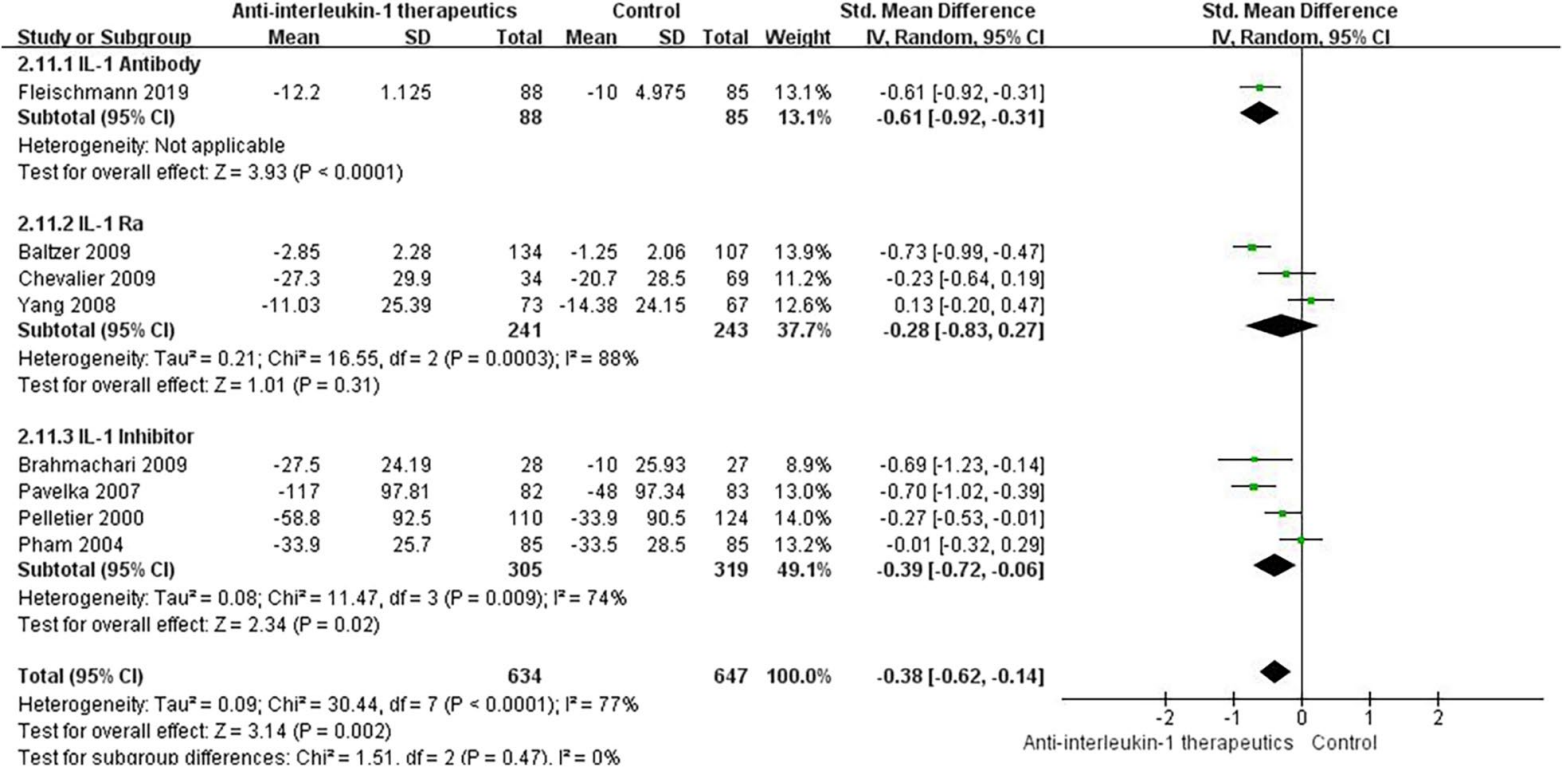
Knee pain score results by subgroup

Following anti-IL-1 or control treatment, four studies [51, 53, 60, 62] assessed pain scores with the WOMAC, and four studies [54, 56, 59, 61] assessed pain scores with the VAS. We found a statistically significant pain decrease in the anti-IL-1 therapeutic group compared with the control group (SMD =  − 0.38, 95% CI: − 0.62 to **-**0.14; *p* < 0.001; *I*^2^ = 77%, Fig. 3). The details of the subgroup analyses are presented in Fig. 4.

### Knee function scores

**Fig. 5:**
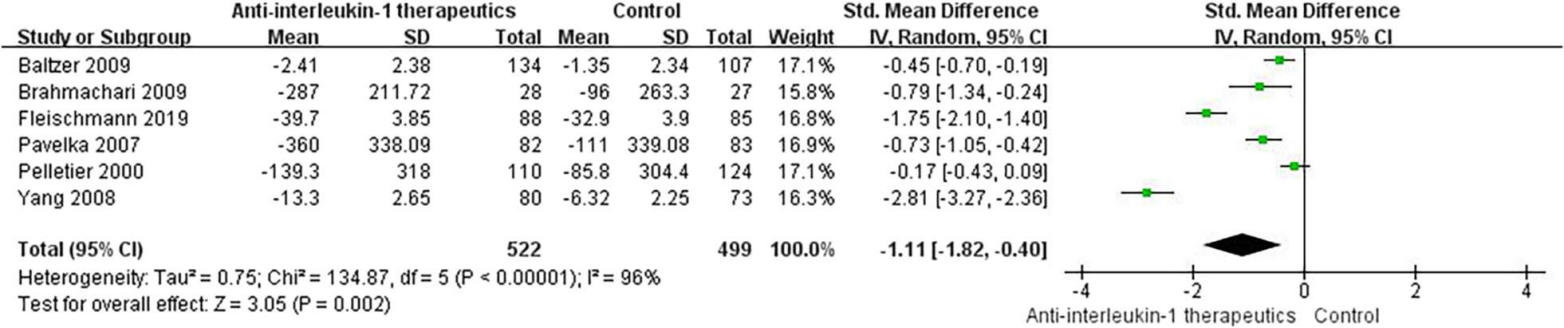
Knee function score results

**Fig. 6:**
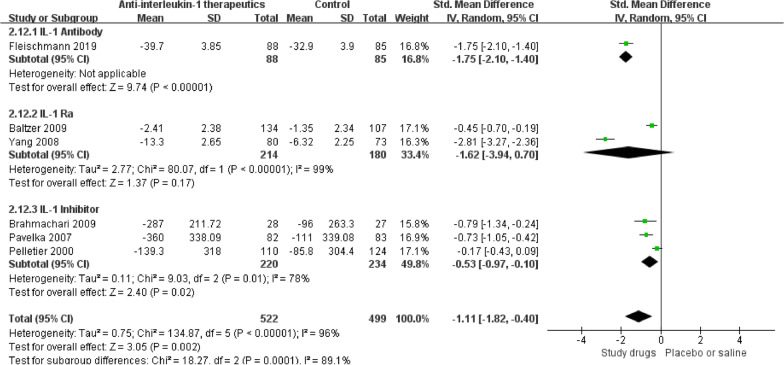
Knee function score results by subgroup

Following anti-IL-1 or control treatment, five studies [51, 53, 54, 59, 60, 62] assessed function scores with the WOMAC, and one study [54] assessed function scores with the KOOS. Significant improvement in knee function was found in the anti-IL-1 therapeutic group compared with the control group (SMD =  − 1.11, 95% CI: − 1.82 to-0.40; *p* = 0.002; *I*^2^ = 96%, Fig. 5). Details of the subgroup analyses are presented in Fig. 6.

### Safety of biological agents in the treatment of OA

#### Any AE

**Fig. 7:**
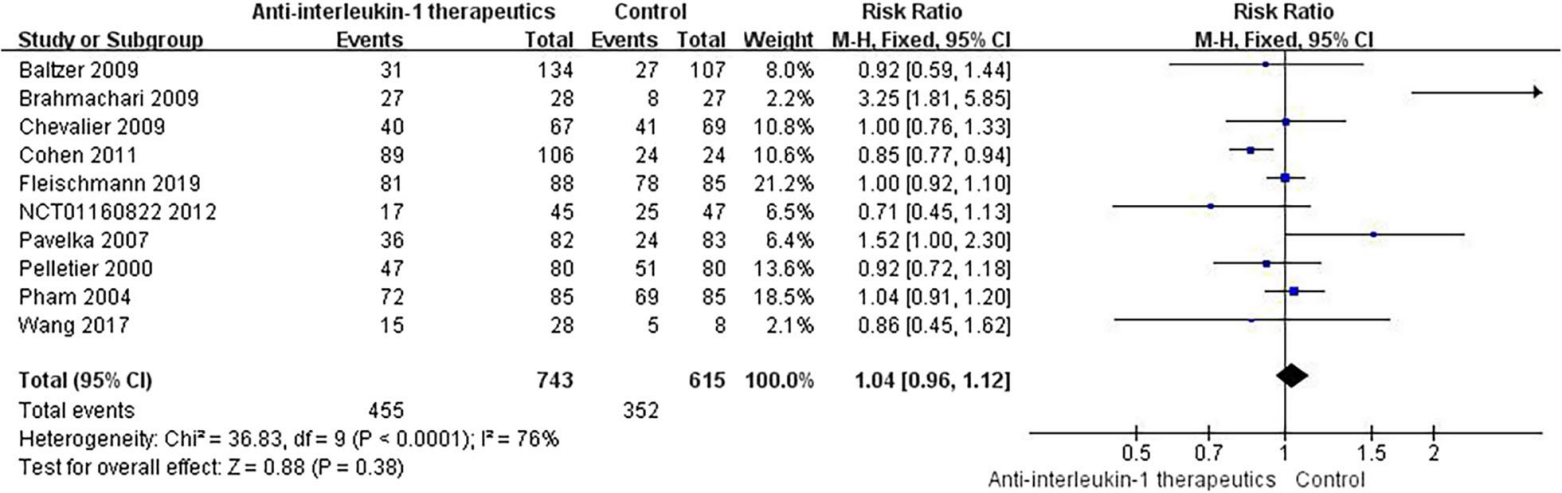
Results for any AE

**Fig. 8:**
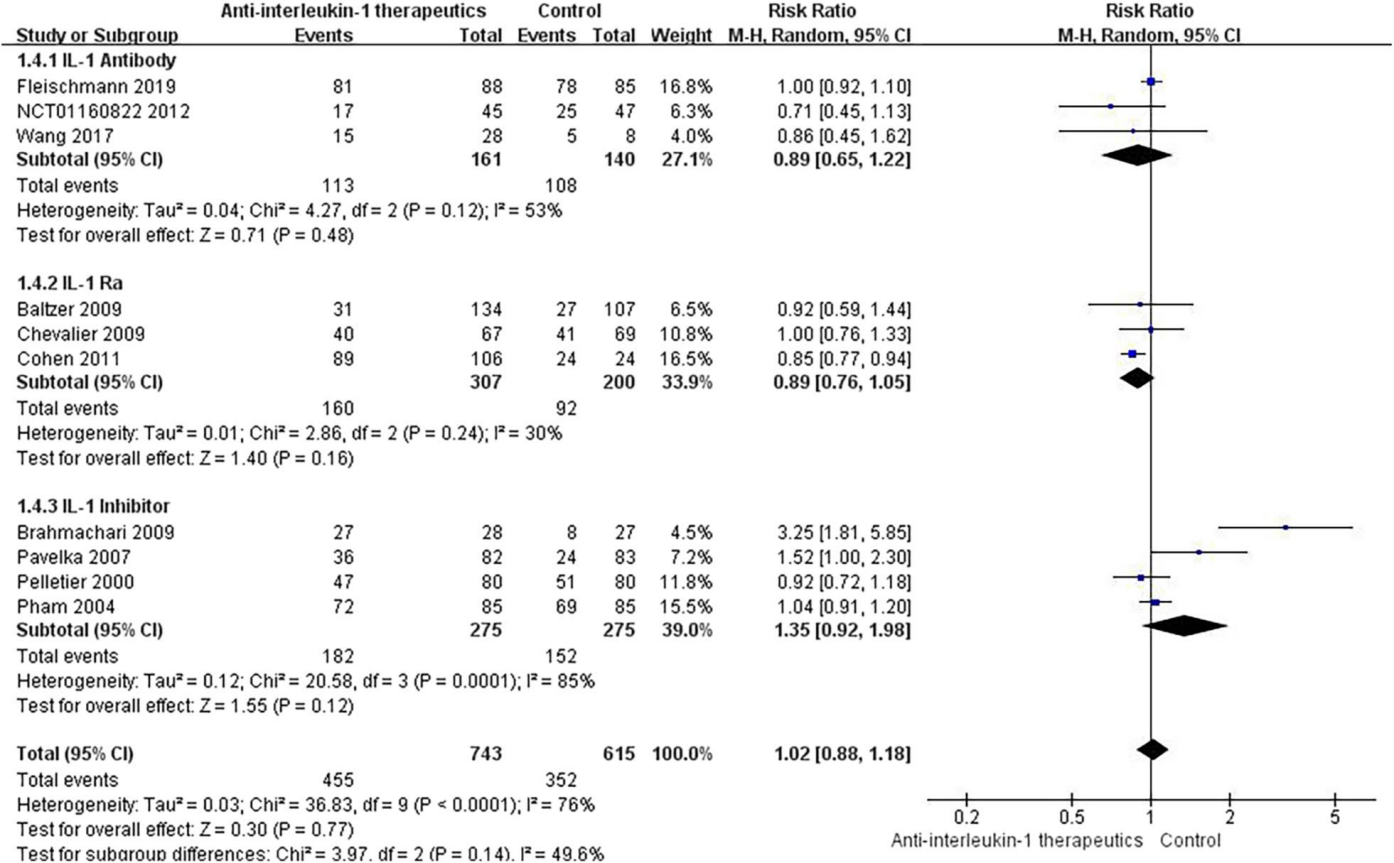
Subgroup results for any AE

A total of 11 studies provided data on the incidence of any AE. Among all AEs, infections, injection site reactions and neutropenia were commonly observed in patients treated with IL-1 antibodies. Headache and upper respiratory tract infections were more frequent in OA patients treated with IL-1 Ras. More patients treated with the IL-1 inhibitor had knee pain, respiratory system disorders, diarrhoea, skin disorders, and gastrointestinal disorders. Overall, the incidence of any AE was significantly different between the anti-IL-1 therapeutic group and the placebo group (RR = 1.02, 95% CI = 0.88–1.18, *p* < 0.001, *I*2 = 76%) (Fig. 7). Details of the subgroup analyses are presented in Fig. 8.

#### Serious AEs

**Fig. 9:**
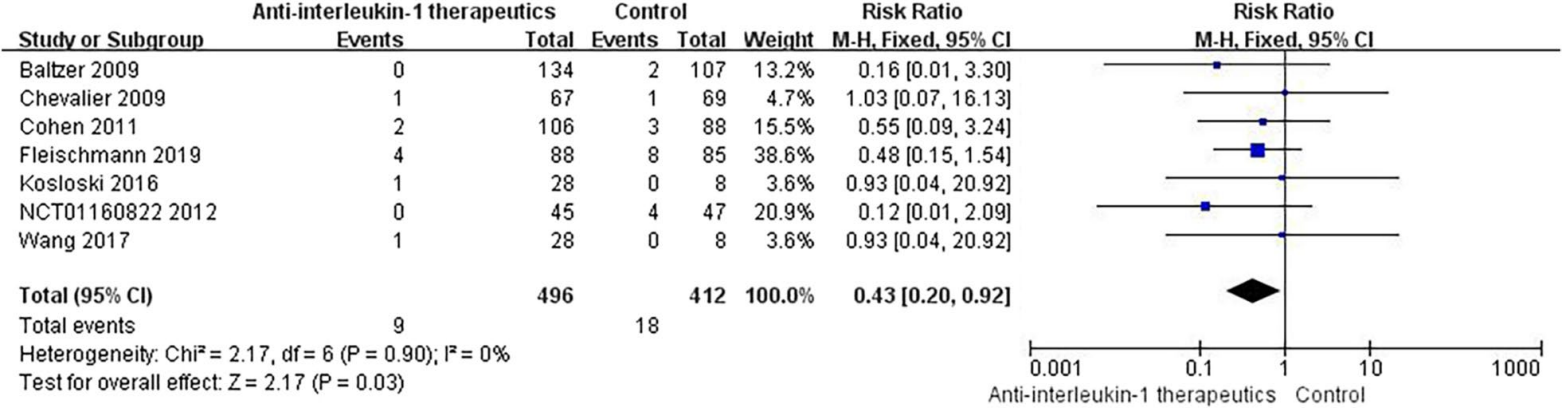
Results for SAEs

**Fig. 10:**
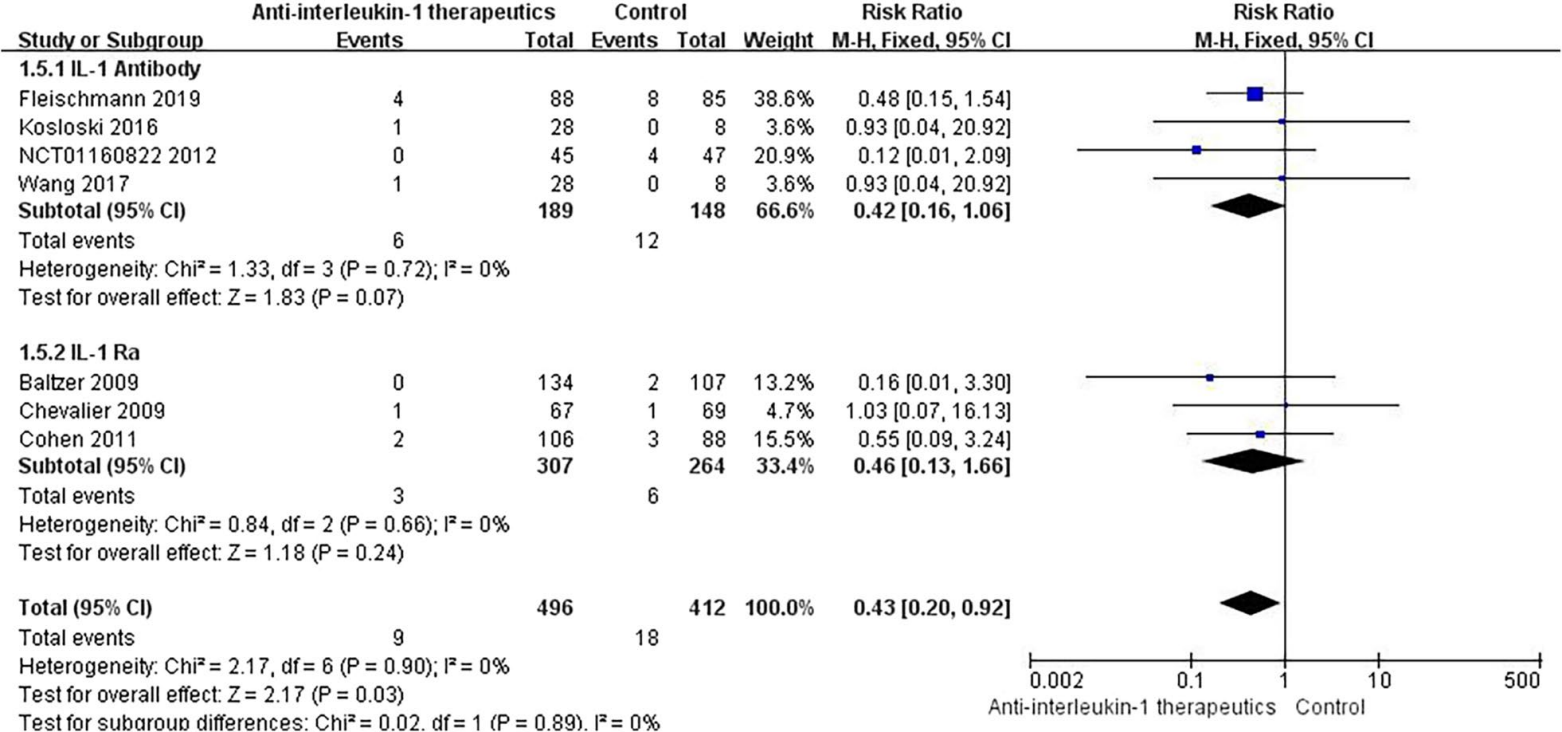
Subgroup results for SAEs

The SAEs in patients treated with IL-1 Ras included haemorrhagic diarrhoea, pneumonia, pancreatitis, and Staphylococcus infection. Serious infection, malignancy, fracture, and injury were observed in RCTs of IL-1 antibodies, but no serious complications were reported with IL-1 inhibitor therapy. Notably, no significant difference was found between the anti-IL-1 therapeutic and placebo groups in terms of the incidence of SAEs (RR = 0.43, 95% CI = 0.20–0.92, *p* = 0.90, *I*^2^ = 0%) (Fig. 9). Compared with placebo, neither IL-1 Ras nor IL-1 antibodies were associated with any significantly increased incidence of SAEs (Fig. 10).

#### Discontinuation due to AEs

**Fig. 11:**
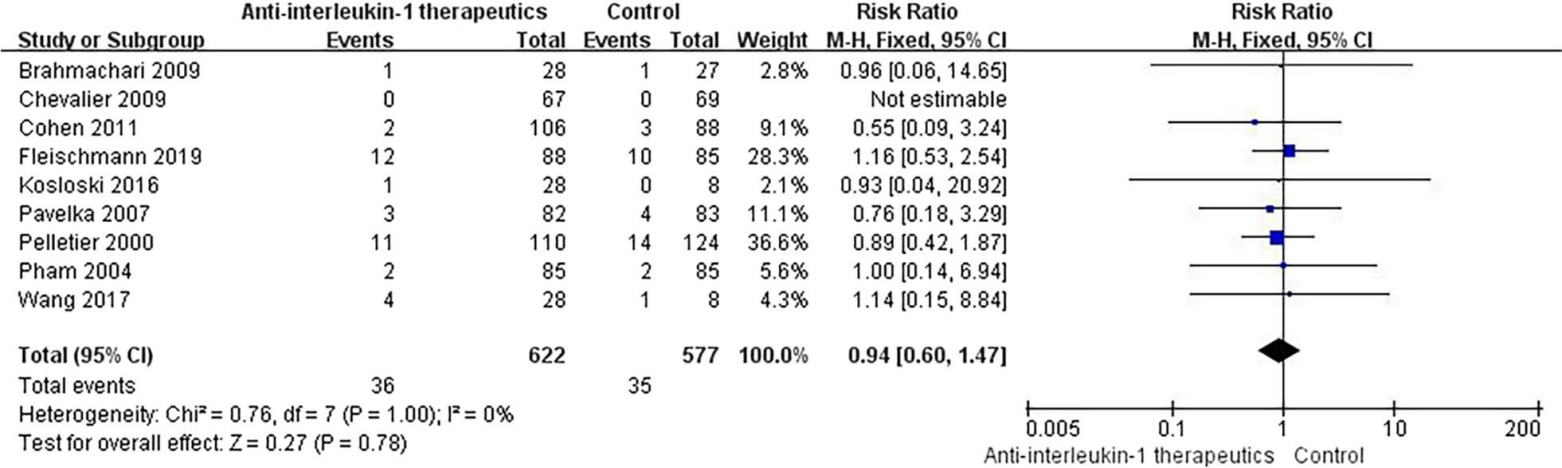
Results for discontinuations due to AEs

**Fig. 12:**
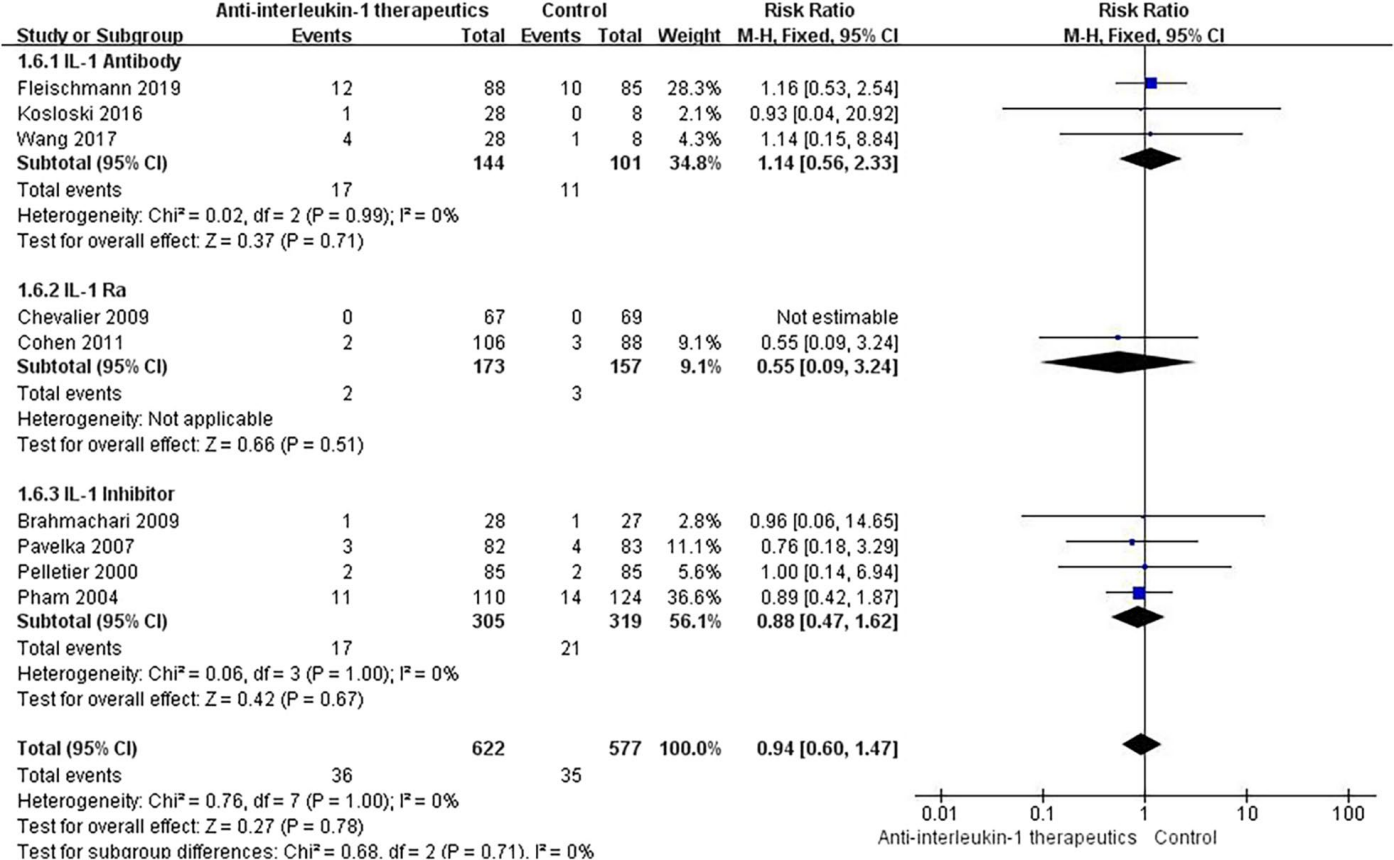
Subgroup results for discontinuations due to AEs

The number of patients discontinued due to AEs was extracted from 9 studies with the data available. No significant difference was found in the incidence of discontinuations due to AEs between the experimental groups and the control group (RR = 0.94, 95% CI = 0.60–1.47, *p* = 1.00, *I*^2^ = 0%) (Fig. 11). Compared with placebo, none of the three types of anti-IL-1 therapeutics were associated with any significantly increased incidence of discontinuations due to AEs (Fig. 12).

## Discussion

This meta-analysis comprehensively investigated the efficacy and safety of anti-IL-1 therapeutics, including IL-1 antibodies, an IL-1 inhibitor, and IL-1 Ras, in patients with KOA. The pooled results indicated that anti-IL-1 therapeutics were significantly superior to placebo in terms of pain relief and functional improvement. The incidence of any AE was higher following treatment with anti-IL-1 therapeutics; however, no significant difference in SAEs or discontinuations due to AEs was found compared with placebo. Subgroup analyses showed that IL-1 antibodies and the IL-1 inhibitor provided superior pain relief and functional improvement, whereas IL-1 Ras did not. However, the IL-1 inhibitor increased the incidence of any AE but not of SAEs or discontinuations due to AEs. IL-1 antibodies and IL-1 Ras showed no difference in safety compared with placebo.

To update the anti-IL-1 therapeutic evidence in the treatment of KOA, we included 12 RCTs covering three anti-IL-1 therapeutic categories based on the mechanism of action: ABT981, AMH108, and canakinumab were reported in four studies as IL-1 antibodies [51, 55, 57, 58]; Orthokine and Anakinra were reported in four studies as IL-1 Ras [52–54, 56]; and diacerein was reported in four studies as an IL-1 inhibitor [59–62]. Compared with previous studies that were either narrative reviews or meta-analyses involving only some anti-IL-1 therapeutics [46–48], the present work comprehensively evaluated the efficacy and safety of the three main anti-IL-1 therapeutics, including IL-1 antibodies, IL-1 Ras, and an IL-1 inhibitor. Considering that all of the included trials were double-blinded randomized placebo-controlled trials, our subgroup analyses according to the mechanism of action enabled indirect comparisons for these three main anti-IL-1 therapeutic categories.

The antagonism of IL-1 in the treatment of OA as well as the potential pathways has been continuously discovered [63–65]. The study conducted by Chevalier [66] indicated that IL-1 can increase the production of matrix metalloproteinase (MMP) and inhibit the synthesis of type II collagen and proteoglycans. MMP is one of the major enzymes in the degradation of cartilage extracellular matrix components, and type II collagen and proteoglycans are important intermediate substances that can promote chondrocyte differentiation. Honourati et al*.* reported that IL1-β can enhance vascular endothelial growth factor (VEGF) secretion to varying degrees through dedifferentiated OA chondrocytes. Several studies have shown that reducing IL-1 by different means can inhibit the inflammatory response caused by IL-1 in human OA chondrocytes [67–70]. Therefore, the IL-1 pathway is a promising target for the treatment of patients with OA. The three types of anti-interleukin-1 therapeutics, including IL-1 antibodies, IL-1 Ras, and IL-1 inhibitors, are all therapeutic agents that interfere with the IL-1 pathway. IL-1 antibodies are a kind of therapeutic human dual variable domain immunoglobulins capable of potently neutralizing human IL-1α and/or IL-1β [71]. The interleukin-1 receptor antagonist (IL-1Ra) is a member of the IL-1 family that binds to IL-1 receptors, which is an important anti-inflammatory protein in arthritis [72]. IL-1 inhibitor is defined as a kind of purified compound which can inhibit the production and activity of interleukin 1 [61].

Since different scales (the WOMAC and VAS for pain evaluation, the WOMAC and KOOS scales for function evaluation) were used in the studies included, we calculated the SMD for each study using Cohen’s d method. According to our results, anti-IL-1 therapeutics provided statistically significant effects on pain relief and functional improvement. The results of subgroup analyses according to the mechanism of action showed that IL-1 antibodies and the IL-1 inhibitor were both associated with significantly higher pain relief and functional improvement than placebo, but IL-1 Ras were not. Several studies have been reported to evaluate the efficacy of anti-IL-1 therapeutics in KOA. A systematic review indicated that IL-1 Ra may be an effective adjunct for those unresponsive to traditional intra-articular therapies [46], which is consistent with our results. However, another meta-analysis indicated that IL-1 antibodies led to no improvement in pain or function compared to placebo [48]. The number of research articles on anti-IL-1 therapeutics in KOA is not enough to reach a consensus on the efficacy of IL for KOA, and more randomized controlled trials and meta-analysis are necessary to update the anti-IL-1 therapeutic evidence in the treatment of KOA.

With respect to the safety of anti-IL-1 therapeutics, the results of further subgroup analyses showed that the IL-1 inhibitor was associated with a higher incidence of any AE, whereas IL-1 antibodies and IL-1 Ras were not. The common AEs in the treatment of IL-1 inhibitors were pain, respiratory system disorders, diarrhoea, skin disorders, and gastrointestinal disorders. These adverse events are self-limited and can resolve following adequate rest. According to the results of the subgroup analyses, IL-1 antibodies, IL-1 Ras, and the IL-1 inhibitor were not associated with a significant difference in SAEs or discontinuations due to AEs compared with placebo. Because of the small number of studies included, we did not perform a further analysis on the effect of different interventions on any AE, SAEs, or discontinuations due to AEs. Therefore, the safety of the IL-1 inhibitor reveals the need for further investigations and great caution in upcoming trials. Contrary to the IL-1 inhibitor, IL-1 antibodies and IL-1 Ras showed favourable tolerability in the treatment of KOA, but attention should still be given to the risk of infection, even if there are no safety concerns.

There is substantial heterogeneity surrounding the treatment effects reported, even after splitting the analyses in subgroups. We consider several reasons to explain this phenomenon. First, different medicines were used in different studies even in the same subgroup (Table.1). IL-1 antibodies included two medicines: ABT981, AMH108, and canakinumab. IL-1 Ras included three medicines: AMG 108, Orthokine, and Anakinra. Second, dosing, method, and/or frequency of administration were inconsistent (Table 1). Dosing varied from 25 to 600 mg. Subcutaneous injection, intravenous injection, and oral were applied in different studies. Frequency varied from twice a day to once every 4 weeks. Third, the pain and function outcome were assessed by different scales: the WOMAC and VAS for pain evaluation, the WOMAC and KOOS scales for function evaluation. Besides, baseline characteristics of the included patients in the same subgroups were also inconsistent. All of above reasons may contribute to substantial heterogeneity after splitting the analyses in subgroups. But limited by the small number of studies included, we could not perform a further subgroups analysis for above factors.

This study has several strengths. Our extensive literature search makes it seems unlikely to miss the clinical RCT associated with this study and the latest RCT for inclusion in the field to date. Trial selection and data extraction, including quality assessment, were performed independently by 2 authors and were discussed with a third senior orthopaedic specialist, thus minimizing bias and the occurrence of transcriptional errors. The highlight is that, unlike other meta-analyses that included studies about only one of the mechanism of anti-IL-1 therapeutics, our study included the latest RCTs about three mechanisms of anti-IL-1 therapeutics, thus providing the most comprehensive update on the effectiveness and safety of anti-IL-1 therapeutics for the treatment of KOA. Only RCTs were included; therefore, by excluding observational studies, we removed the inherent selection bias associated with that study design. A detailed assessment of the methodological quality of the included studies was performed. In addition, we performed subgroup analyses according to the different mechanisms of action of anti-IL-1 therapeutics, thus observing the effect of the IL-1 inhibitor, IL-1 antibodies, and IL-1 Ras on the target outcome separately and overcoming the limitations of the previous systematic evaluation.

### Limitations

This study has several limitations. First, similar to most systematic evaluations, our study was limited by the quality of the included RCTs. Most trials were of poor methodological quality or showed selective reporting. Only four trials [54, 56, 58, 59] described the method of allocation concealment. The potential risk of bias weakened our ability to draw conclusions regarding the treatment effects. Second, none of the included studies reported on knee survivorship, that is, the number of patients for whom anti-IL-1 therapeutics ultimately failed and thus went on to undergo total knee arthroplasty. Third, the studies included were heterogeneous in terms of dosage and intervention, which are factors that may lead to differing biological activity of anti-IL-1 therapeutics and thus different physiological responses in patients. Additionally, the follow-up time among the included studies also varied, ranging from 12 to 52 weeks. Furthermore, there was no publication bias in this study. The authors had considered assessing publication bias by funnel plot once but we had not done it finally. As Sterne JAC, Sutton A J et al. thought, tests for funnel plot asymmetry should not be used when there are fewer than 10 studies in the meta-analysis because test power is usually too low to distinguish chance from real asymmetry [73]. There are only eight studies with knee pain outcome and only five studies with knee function score in our meta-analysis. These factors weakened our ability to draw conclusions on the effect of anti-IL-1 therapeutics compared with control treatment for KOA.

## Conclusions

Our study updates the anti-IL-1 therapeutic evidence in the treatment of KOA. Anti-interleukin-1 therapeutics could relieve OA-related pain and improve function, but is probably associated with an increased risk of adverse events. Specially, the efficacy and safety of anti-IL-1 therapeutics varied according to the mechanism of action. IL-1 antibodies and an IL-1 inhibitor could relieve OA-related pain and improve function, whereas IL-1 Ras could not. IL-1 antibodies and IL-1 Ras were relatively safe options, but IL-1 inhibitors were associated with safety concerns. Due to the low quality of the studies and the limited data currently available, more high-quality RCTs are needed.

## Data Availability

The data that support the findings of this study are available from the corresponding author upon reasonable request.

## Abbreviations

IL-1 Ra: Interleukin-1 receptor antagonists
KOA: Knee osteoarthritis
RCT: Randomized controlled trial
AE: Adverse effect
SMD: Standardized mean difference
RR: Risk ratio
SAE: Serious adverse effect
CI: Confidence interval
OA: Osteoarthritis
NSAIDs: Nonsteroidal anti-inflammatory drugs
HA: Hyaluronic acid
IL: Interleukin
TNF: Tumour necrosis factor
NGF: Nerve growth factor
PRISMA: Preferred Reporting Items for Systematic Reviews and Meta-Analyses
MeSH: Medical Subject Headings
WOMAC: Western Ontario and McMaster Universities Osteoarthritis Index
VAS: Visual analogue scale
KOOS: Knee Injury and Osteoarthritis Outcome Score
BMI: Body mass index
I-V: Inverse-variance
